# Sporadic Creutzfeldt–Jakob Disease in the young (50 and below): 10-year review of United Kingdom surveillance

**DOI:** 10.1007/s00415-022-11467-3

**Published:** 2022-11-05

**Authors:** Johnny Tam, John Centola, Hatice Kurudzhu, Neil Watson, Janet MacKenzie, Margaret Leitch, Terri Hughes, Alison Green, David Summers, Marcelo Barria, Colin Smith, Suvankar Pal

**Affiliations:** National CJD Research & Surveillance Unit (NCJDRSU), Centre for Clinical Brain Sciences, Chancellor’s Building, University of Edinburgh, Edinburgh, Scotland, UK

**Keywords:** Creutzfeldt–Jakob Disease, Sporadic, Prion, Young, Surveillance

## Abstract

**Introduction:**

Sporadic Creutzfeldt–Jakob Disease (sCJD) is the commonest human prion disease, with a median age of onset of 68 years. We characterise the clinical, investigation, and neuropathological features in young individuals with sCJD using data from UK national CJD surveillance.

**Methods:**

Referrals between 2011 and 2021 were examined, with definite (post-mortem confirmed) or probable sCJD cases included. Clinical features, MRI, EEG, CSF RT-QuIC, 14-3-3, *PRNP* sequencing and neuropathological findings were examined. We compared younger (≤ 50 years age of onset) with older individuals. Records of Non-sCJD referrals were also reviewed.

**Results:**

46 (4%) young individuals were identified (age at onset 25–50) from 1178 cases. 15 (33%) were autopsy confirmed. Psychiatric disturbance (37% vs 22%, *p* = 0.02) and headache (11% vs 3%, *p* = 0.01) at presentation, and longer disease duration (by 1.45 months, 95% CI 0.43–2.79, logrank *p* = 0.007) were commoner. CSF RT-QuIC showed lower sensitivity (82% vs 93%, *p* = 0.02). There was no difference in sensitivity of MR brain or CSF 14-3-3. There were no significant co-pathologies in autopsy-confirmed cases. For non-sCJD referrals, 41 cases were of other CJD subtypes, and 7 non-prion diagnoses.

**Conclusions:**

Young-onset sCJD is more likely to present with neuropsychiatric symptoms and headache, longer disease duration, and lower sensitivity of RT-QuIC. These findings may be driven by the underlying molecular subtypes. Our results guide the evaluation of younger individuals presenting with rapidly progressive cognitive, neuropsychiatric, and motor decline, and emphasise the need for additional vigilance for atypical features by clinicians and CJD surveillance programmes worldwide.

**Supplementary Information:**

The online version contains supplementary material available at 10.1007/s00415-022-11467-3.

## Introduction

### Background

Creutzfeldt–Jakob Disease (CJD) is a fatal human prion disease, characterised by rapidly progressive cognitive, neuropsychiatric, and motor impairment. Sporadic CJD (sCJD) is the most common aetiology (85%), with a worldwide annual incidence of 1–2 per million [1, 2]. Other aetiologies include inherited prion disease and acquired subtypes such as variant (vCJD) and iatrogenic CJD (iCJD). Prion diseases are characterised by the accumulation and propagation of transmissible misfolded proteins [3], with tissue distribution varying between disease aetiologies which poses significant infection control and public health risks [4]. This is epitomised by the vCJD epidemic of the 1990s–2000s, causally linked to bovine spongiform encephalopathy (BSE) in cattle [5, 6]. Continued international surveillance of human prion diseases is important for the implementation of appropriate public health measures and ascertainment of further cases of acquired CJD, as well as vigilance for novel human prion diseases [7].

sCJD occurs most commonly in the sixth and seventh decades of life [1], but can also affect much younger individuals. This often poses significant challenges for the diagnosis and management of younger individuals, including differentiation from other aetiologies of CJD, particularly genetic and variant CJD. In the last decade there have been significant advances in diagnostic investigations of sCJD; specifically the increased use of diffusion-weighted magnetic resonance imaging (MRI) [8], and the development of the highly specific real-time quaking-induced conversion assay (RT-QuIC) [9]. These advances have helped improve case ascertainment in sCJD, especially amongst younger individuals. Despite these developments, understanding of the clinical features, investigation results, and neuropathological findings of younger individuals with sCJD remain sparse, largely confined to descriptions from small case series [10–13] and isolated case reports [14–17]. An updated study is required to characterise this group of individuals, as these previous studies largely predated the widespread use of currently available highly sensitive investigations in CJD.

### Objectives

We interrogated retrospective data from the UK National CJD Research and Surveillance Unit programme to comprehensively describe the clinical features, investigation results, prion protein genotyping and neuropathological findings of young individuals diagnosed with sCJD. Given the diagnostic challenge in young individuals with suspected prion disease, we also reported on referrals where a different prion disease subtype or a non-prion disease was eventually diagnosed. We discuss our findings in the context of the established literature in the area and provide recommendations for diagnostic assessment of young individuals suspected of having sCJD, including significant differences compared to important differential diagnoses such as vCJD and inherited prion disease.

## Methods

This report follows the Strengthening the Reporting of Observational Studies in Epidemiology (STROBE) guidelines [18].

### UK national CJD surveillance

Surveillance data ascertained by the UK National CJD Research and Surveillance Unit (NCJDRSU) was used in this study. The UK NCJDRSU has demonstrated extremely high rates of case ascertainment linked to detailed clinical phenotyping using established assessment protocols [19]. Data collected as part of UK CJD surveillance has been approved by a research ethics committee review as essential for public health purposes.

### Sporadic CJD cohort

We extracted clinical data from all referrals in a ten-year period between 31st August 2011 and 31st August 2021. All cases of probable and definite sCJD were examined, as defined by the International CJD Surveillance Network diagnostic criteria [20, 21]. The criteria were updated and adopted from 1st January 2017 [20]. Prior to this, cases were classified according to diagnostic criteria from 2010 [21].

We identified a group of young individuals who were diagnosed with sCJD to evaluate differences compared with older individuals. We defined young individuals as those with an age of onset at or lower than 2 standard deviations below the mean age of onset. This was 50 years in our study population, where the mean age of onset was 68 years.

### Clinical data

Individuals with suspected CJD were referred from across the UK to the NCJDRSU for specialist assessment. They were assessed by a physician from the Unit in-person at the referring hospital, at home, or remotely via a telehealth consultation [22]. Clinical history and routine investigation results were obtained from the patient, their relatives, and hospital notes. Clinical data was then entered into a standardised research questionnaire and stored in the NCJDRSU database.

Disease duration was measured as the number of months elapsed between symptom onset and death. Symptom onset was defined as the first date on which a symptom ascribed to the illness was manifested. Diagnostic latency was measured as the months elapsed between symptom onset and the date of diagnosis. Date of diagnosis was defined as the first date on which a diagnosis of sCJD was first made, including post-mortem diagnosis.

Presenting symptoms at the onset of disease were classified according to one of 14 symptom complexes (psychiatric & behavioural disturbance, cognitive impairment, motor and gait abnormalities, visual disturbances, headache, sleep disturbance, dizziness and vertigo, fatigue and malaise, sensory disturbance, speech disturbance, language disturbance, auditory disturbance, seizures and other). Presenting symptom category definitions are summarised in Supplement 1. Symptoms present at the point of diagnosis were also studied.

### Magnetic resonance imaging and electroencephalogram

MRI brain studies were transferred to the NCJDRSU from referring hospitals for assessment by neuroradiologists with a specialist interest in prion disease (D.S.). T2-weighted, fluid-attenuated inversion recovery (FLAIR), and diffusion-weighted imaging (DWI) sequences were routinely analysed for cortical, basal ganglia and thalamic signal abnormalities suggestive of sCJD [8]. An overall impression for sCJD (positive, equivocal, or negative with reference to diagnostic criteria) was provided.

Where an electroencephalogram (EEG) was performed at the referring hospital, the written report was reviewed by an NCJDRSU physician. EEGs reported to show abnormal periodic sharp wave complexes suggestive of sCJD were reviewed [23].

### Cerebrospinal fluid analysis

Cerebrospinal fluid (CSF) was obtained by a lumbar puncture at the referring hospital and transferred to the NCJDRSU CSF laboratory for testing of RT-QuIC and 14-3-3. Results for RT-QuIC were reported as either positive or negative. 14-3-3 was reported as positive, weak positive or negative. For this study, weak positive 14-3-3 results were regarded as negative.

### Prion protein gene *(PRNP)* sequencing

Blood samples were obtained in individuals where consent was given for prion protein gene *(PRNP)* mutation testing. Individuals with a pathogenic mutation and diagnosed with inherited prion disease were excluded from the study. A polymorphism at codon 129 of the *PRNP* gene was recorded as homozygous for methionine (MM) or valine (VV), or heterozygous (MV) genotypes.

### Neuropathological, biochemical classification and molecular subtypes

In patients who underwent autopsy, definite sporadic CJD was confirmed by neuropathological examination. Macroscopic and microscopic examinations were conducted. Prion pathology was identified using immunohistochemistry with 12F10 and KG9. Other neurodegenerative co-pathologies were screened for with β-amyloid, tau and pTDP-43 immunohistochemistry. Proteinase K treatment and western blotting in frozen brain material identified the biochemical classification of abnormal prion protein (PrP^res^) as type 1 (21 kDa) or type 2 (19 kDa). Cases with a mixture of abnormal prion protein types or low molecular weight (LMWt) bands were also reported. Codon 129 polymorphism and PrP^res^ biochemical subtype were combined to provide a molecular subtype of disease [24].

### Non-sporadic CJD referrals

For qualitative comparison, we extracted information from other referrals to the NCJDRSU in the same ten-year period and age cut-off. Cases of other prion disease subtypes were included (including inherited prion disease, vCJD and iCJD), as well as cases where a non-prion diagnosis was assessed to be more likely.

### Addressing potential biases

Case selection was based on comprehensive surveillance for CJD in the United Kingdom, which minimises selection bias of study participants [25]. Performance bias is limited as all cases are assessed using a standardised research questionnaire and classified according to the 2010 or 2017 international CJD surveillance diagnostic criteria [19, 25]. All pre-specified outcomes are reported, along with the proportion of available or missing data.

### Statistical analysis

RStudio 1.4.1717 (RStudio Team)[26] was used for statistical calculations and the production of figures. Missing entries were omitted from the analysis. We elected not to conduct a statistical comparison where there was a large proportion of missing data. Categorical variables were compared using chi-squared test, Fisher’s exact test, or exact multinomial test. Parametric continuous data were compared using analysis of variance (ANOVA). Time-to-event data such as disease duration and diagnostic latency were analysed by the Kaplan–Meier method and Wilcoxon rank sum test [27, 28].

## Results

### Age of onset and basic demographics

1178 individuals diagnosed with probable or definite sCJD were identified in the ten-year study period. 46 (4%) individuals were identified to have an age of onset at 50 years or below. 15 were neuropathologically confirmed definite sCJD, with the remaining 32 classified as probable sCJD. 1 patient was lost to follow-up shortly after diagnosis as they relocated abroad. The proportion between sexes was approximately equal (26 males, 20 females, *p* = 0.47). In this group ethnicity data was missing in 1 individual. 89% were of white European descent (40/45), 3 were of South Asian descent, 1 of African descent and 1 of mixed ethnicity.

### Disease duration

**Fig. 1:**
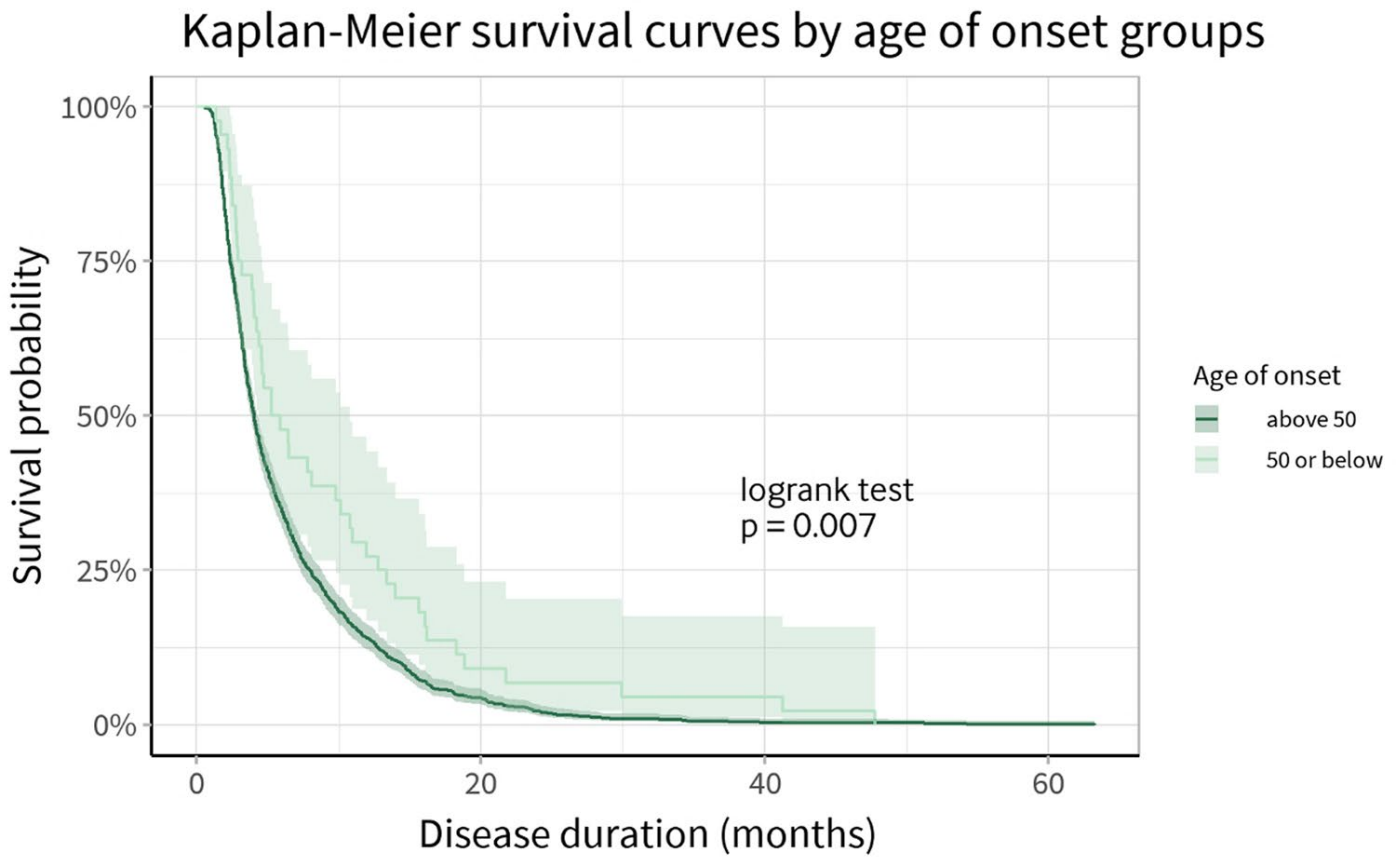
Kaplan–Meier survival curves by age of onset groups with 95% confidence bands. Logrank test finds a significant difference between survival curves

Disease duration data was available in 97% of the study population. There was no missing disease duration data in the young age of onset (≤ 50 years) group, where the median duration of illness was 5.59 (range 1.35–47.80) months. Survival analysis using the Kaplan–Meier method showed disease duration was significantly different between these groups (logrank *p* = 0.007) (Fig. 1). Median disease duration in the young age of onset group was longer by 1.45 (95% CI 0.43–2.79) months compared to older individuals.

### Diagnostic latency

Diagnostic latency data were missing in only 2% across the study population. In the young age of onset (≤ 50 years) group median time to diagnosis was 4.80 (range 1.17–35.05) months, which was not significantly different compared to older individuals when analysed by the Kaplan–Meier method and logrank test (*p* = 0.20).

### Symptoms

**Fig. 2:**
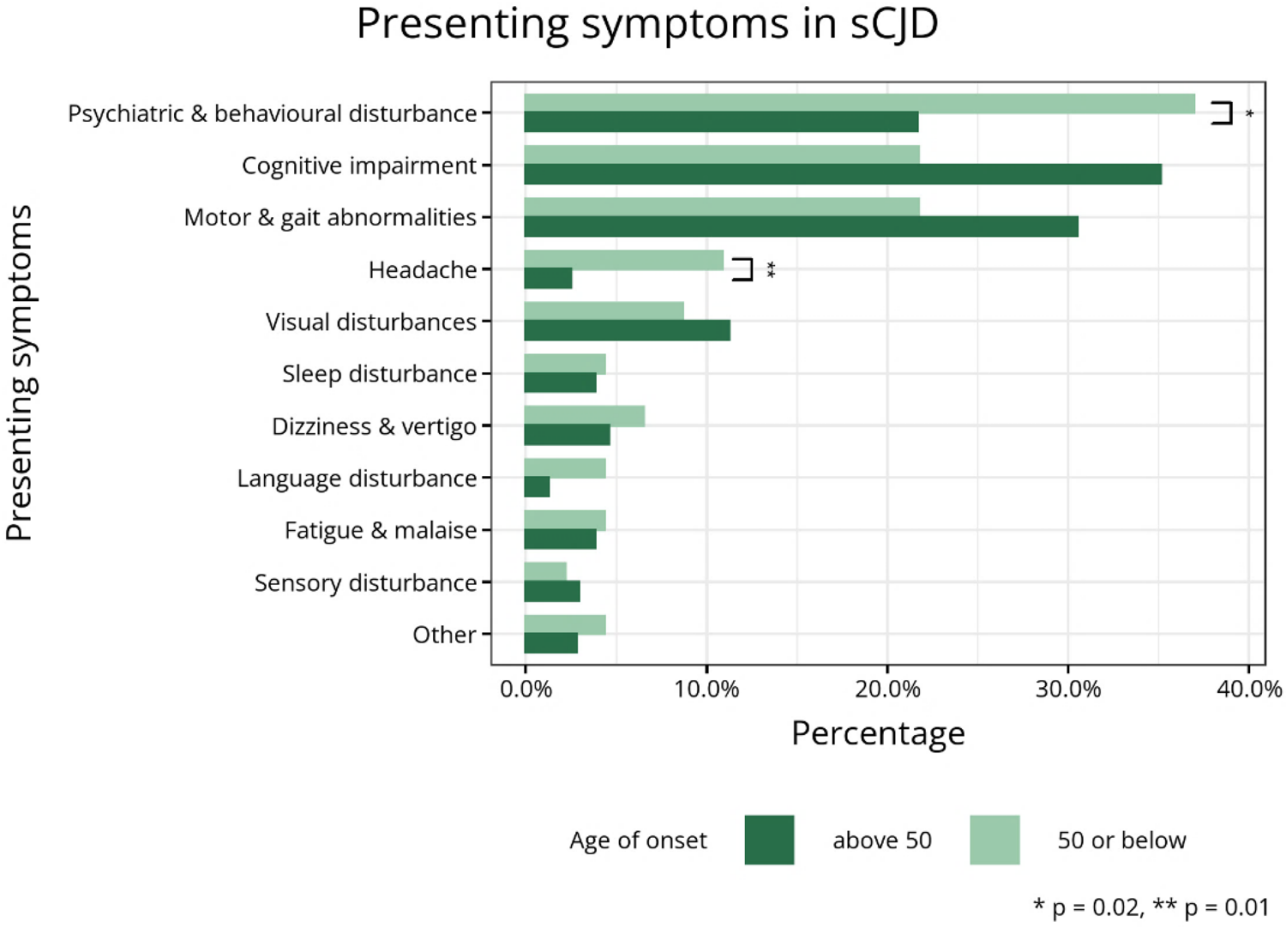
Bar plot of presenting symptom categories in sCJD by age of onset groups (definitions in Supplement 1). *p-value determined by *X*^2^ normal approximation. **p-value determined by Fisher’s exact test due to small expected frequencies

Presenting symptom data were missing in 8% across the study population. The most common presenting symptom category in the young age of onset group (≤ 50 years) was psychiatric and behaviour disturbance (17/46, 37%), which was higher in proportion than in older individuals (vs 22%, *p* = 0.02). Headache also appeared commoner in the younger age group (11% 5/46 vs 3%, *p* = 0.01). Review of case notes showed that only 1 individual had a known previous history of headaches or migraines. Differences in the proportions of other presenting symptom categories were not statistically significant (Fig. 2).

**Fig. 3:**
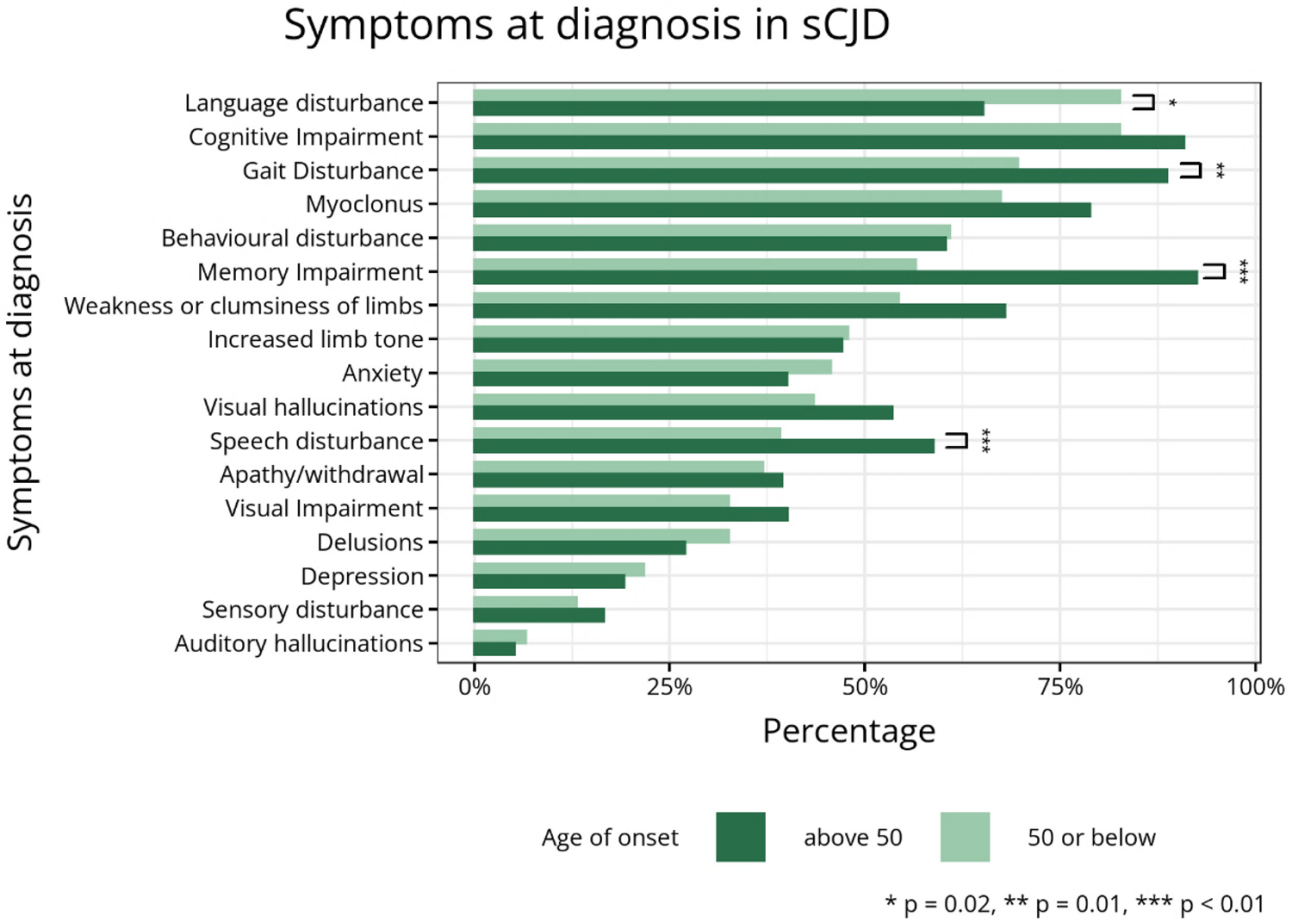
Barplot showing symptoms present at the point of sCJD diagnosis. *,**,***p-values determined by *X*^2^ test between age of onset groups

Data on symptoms present at the point of diagnosis was available in 95% of the study population. A higher proportion of individuals in the young age of onset group had language disturbance, such as expressive dysphasia (85% vs 65%, *p* = 0.02). Conversely, a smaller proportion of individuals in this group had gait disturbance (70% vs 89%, *p* < 0.01), memory impairment (57% vs 92%, *p* < 0.01) and speech disturbance including dysarthria and dysphonia (39% vs 59%, *p* = 0.01) compared to older individuals (Fig. 3).

### MRI Brain and EEG

All 46 individuals in the young age of onset (≤ 50 years) group had MRI brain studies. An MRI diagnostic for sCJD (‘positive’) was identified in 41/46 (89%) patients. The remaining 5 had suspicious (3), inadequate (1), or negative (1) imaging. This diagnostic sensitivity of MRI brain was the same as in older individuals (804/906, 89%).

Both cortical ribboning and basal ganglia changes were identified in most individuals (36/46, 78%). Isolated cortical ribboning with no basal ganglia signal change was seen in 5 (11%), whilst isolated basal ganglia changes were present in 4 (9%).

An EEG was undertaken in 40/46 (87%) individuals in the young age of onset group. Changes suggestive of sCJD were identified in only 7/40 (18%) individuals. Comparison with older individuals was not performed due to the significant amount of missing data in the older group.

### CSF analysis

The sensitivities of diagnostic CSF tests between the young age of onset (≤ 50 years) group and older individuals are summarised in Table 1. The proportion of younger individuals with positive RT-QuIC was significantly lower than in older individuals (*p* = 0.02). There was no significant difference in the sensitivities of 14-3-3 between groups.

**Table 1:** Comparison of diagnostic CSF test sensitivities in sCJD by age onset groups

Comparison of diagnostic CSF test sensitivities in sCJD		
Age of onset groups	RT-QuIC	14–3-3
≤ 50	82% (30/37)	68% (26/39)
> 50	93% (806/869)	67% (575/860)
*p*-value^a^	*p* = 0.02	*p* = 1.00

^a^*p*-values determined by Fisher’s exact test between the age of onset groups

7 individuals in the young age of onset (≤ 50 years) group had negative RT-QuIC (summarised in Table 2). 4 underwent post-mortem examination where sCJD was confirmed. The remaining 3 were diagnosed as probable sCJD based on radiological features, and vCJD was assessed to be unlikely.

**Table 2:** Investigation findings of 7 young age of onset (≤ 50 years) individuals with negative RT-QuIC

Individuals with negative RT-QuIC in young age of onset (≤ 50 years) group						
Individual	Age of onset	Post-mortem	14-3-3	Codon 129/Molecular subtype	MRI	DWI-MRI findings
1	47	No	+	VV	+	CR -, BG + , T + , P –
2	45	Yes, sCJD confirmed	+	MM1(+ 2)	+	CR + , BG + , T–, P–
3	50	Yes, sCJD confirmed	+	VV1	+	CR + , BG + , T–, P–
4	49	No	–	Missing	+	CR + , BG + , T–, P–
5	26	Yes, sCJD confirmed	+	VV1	+	CR + , BG + , T–, P–
6	51	Yes, sCJD confirmed	-	MM2	+	CR + , BG + , T–, P–
7	49	No	+	MM	+	CR + , BG + , T–, P–

*DWI-MRI* diffusion weight imaging MRI, *CR* cortical ribboning, *BG* basal ganglia, *T* thalamus, *P* pulvinar sign

### *PRNP* gene analysis

Sequencing of the *PRNP* gene was completed in 80% (37/46) of individuals in the young age of onset (≤ 50 years) group with pathogenic mutations excluded. Distribution of codon 129 genotypes between the age of onset groups was not significantly different on *X*^2^ goodness-of-fit test (*p* = 0.52).

*PRNP* gene sequencing results were unavailable in 9 individuals. There was no significant family history of dementia or neurodegenerative conditions in all these cases.

### Neuropathological profiles

Autopsy examination was completed in 15 (33%) of the young age of onset (≤ 50 years) group. This proportion was similar to that in older individuals (33%, 374/1121). Apart from minor Thal stage 1 Alzheimer’s Disease pathology in 4 individuals, there was an absence of neurodegenerative co-pathologies or other neurological diagnoses (Table 3). In the young age of onset (≤ 50 years) group, Western blotting of autopsy brain material was completed in 12 individuals, facilitating biochemical and molecular classification of the disease (Table 2) [24]. 2 VV1 individuals were present amongst only 8 VV1 individuals across the whole study cohort. Given this small sample size, no statistical comparison with older individuals is presented here.

**Table 3:** Neuropathological profiles of sCJD patients with age of onset ≤ 50

Neuropathological profiles of sCJD in age of onset ≤ 50		
Patient	Neurodegenerative co-pathologies	Molecular subtype [24]
1	None	MM1(+ 2)
2	None	VV^a^
3	None	MM1
4	None	MM LMWT(+ 2)
5	None	MM1(+ 2)
6	Thal 1	VV1
7	Thal 1	MM1
8	None	VV1
9	Thal 1	MM2
10	Missing	VV^a^
11	None	MM1(+ 2)
12	None	MV1
13	Thal 1	MM^a^
14	Missing	MV2
15	None	MV1

Thal amyloid stage 1 pathology was identified in 3 patients

^a^Western blotting was not performed.

### Non-sporadic CJD referrals

In total, we identified 48 referrals in the same ten-year period with the age of onset at 50 or below where a different prion disease subtype or a non-prion disease was diagnosed.

41 individuals were diagnosed with a different prion disease subtype. Inherited prion disease was identified in 25 individuals (with disease-causing mutations in the prion protein gene including E200K, P102L, D178N, octapeptide repeat insertion). 14 individuals were diagnosed with iCJD associated with the history of human-derived growth hormone treatment.

Two individuals were diagnosed with vCJD during the study period. Notably, the most recent individual with vCJD presented with a disease course clinically and radiologically mimicking sCJD, before a vCJD diagnosis was confirmed at post-mortem [29]. This individual is also known to be the only neuropathologically confirmed vCJD case known with heterozygosity for methionine and valine at *PRNP* codon 129. The other young individual was diagnosed as probable vCJD based on supportive clinical, neurophysiological, and radiological findings [20]. Both cases had negative CSF RT-QuIC and 14-3-3 analyses.

Seven individuals were identified where prion disease was assessed to be unlikely, where either an alternative neurodegenerative diagnosis was made, or clinical or radiological improvement was observed after referral. CSF diagnostic tests were conducted in six individuals, with negative CSF RT-QuIC analysis in all, and only one individual with positive CSF 14-3-3.

## Discussion

Our study demonstrates that younger individuals with sCJD present differently to older individuals. They are more likely to present with psychiatric disturbance and headaches. At the point of diagnosis younger individuals are more likely to suffer from language disturbance, but less likely to have memory impairment, gait disturbance and speech disturbance. Younger individuals have longer disease duration, but diagnostic latency is not significantly increased in comparison to older individuals. MRI is a sensitive test in young individuals with sCJD, but CSF RT-QuIC appears to have a lower sensitivity. Autopsy revealed only minor Thal amyloid stage 1 co-pathology in 4 younger individuals, with no other significant neurodegenerative co-pathologies.

### Clinical features

The clinical presentation of young-onset sCJD has previously been reported in a meta-analysis by Appleby and colleagues [30]. They found that younger individuals presented more commonly with affective and behavioural symptoms (27.5% and 18.8% respectively, *p* < 0.0001). Other presenting neurological symptoms such as vertigo/dizziness (23%), headache (13%) and myoclonus (11.6%) were also significantly more common in young individuals. As this study was a systematic review of literature, these differences may be confounded by the possibility that more unusual cases of young-onset sCJD would be published in literature. However, the differences in psychiatric illness and headaches are demonstrated in our study and have previously been corroborated by a surveillance-based cohort study of young sCJD individuals with an age of onset below 50, conducted by Boesenberg and colleagues in 2005 [11]. The authors highlighted the possibility that the increased frequency of psychiatric signs may be biased, as they may appear more prominently in younger patients, compared with older patients with a rapidly progressive disease course where other neurological deficits overshadow psychiatric signs and symptoms, limiting detection.

Boesenberg et al. also identified an increased frequency of headaches at disease onset (17% *n* = 9), corroborated in our study. Sample sizes are small in both studies, limiting any statistical conclusions. This difference may simply reflect increased prevalence of headache disorders in younger individuals [31], although only one individual in our group was known to have a history of migraines and headaches.

Our results also showed younger individuals were less likely to have memory impairment, gait disturbance and speech disturbance at the time of diagnosis. This may be due to the better cognitive and functional reserve of younger sCJD patients, which may delay the onset of these symptoms.

Longer disease duration in young-onset sCJD has been well described [10, 11, 32–34], including a European collaborative study by Pocchiari and colleagues in 2004 [33]. Age of onset was reported in 10-year age groups, where 117 individuals with an age of onset at 50 or below were identified in a multicentre cohort of 2304 individuals with sCJD (5%). Median survival was significantly higher in these age groups compared to older individuals (7–58 months). Multivariate analysis showed that increments of 10 years in the age of onset were associated with ~ 30% increase in the risk of death. In Bosenberg and colleagues’ surveillance-based cohort study, median disease duration was reported to be 16 months in patients with an age of onset ≤ 50, 10 months more than in older individuals [11]. In contrast, our study finds a modest but significant increase in median disease duration of 1.45 (95% CI 0.43–2.79) months.

The reasons for this inverse relationship between disease duration and age of onset remain unclear, although several factors have been implicated. Age-related resistance to other causes of death (such as infection) in younger individuals likely plays a significant role. Life-prolonging interventions such as nasogastric feeding and mechanical ventilation[35] may be more frequently offered to younger individuals, especially in the early stages of disease when the diagnosis may be unclear. Increased prevalence of rarer molecular subtypes in younger individuals is also a likely significant factor, especially the VV1 subtype [24].

We did not find a significant age-related difference in diagnostic latency. This provides reassurance that timely diagnosis of sCJD is not significantly affected by age of onset within our surveillance programme.

### Diagnostic investigation sensitivity

Our study supports the high sensitivity of MRI in young-onset sCJD. MRI findings in previous studies lacked DWI sequences in common use now [11], while others were limited by small sample size [12]. Younger individuals exhibit the same typical features of sCJD in older patients, with both cortical ribboning and basal ganglia changes present in most individuals.

The development of highly specific RT-QuIC in CSF has improved accuracy and delays in the diagnosis of sCJD. Previous studies have suggested lower sensitivity in younger sCJD patients [36–38], but how age and other factors affect RT-QuIC positivity remains debated [9, 39].

Our findings add to the current body of evidence for lower RT-QuIC sensitivity in younger individuals. This difference is likely small but clinically relevant for interpreting results in younger individuals. Our findings showed that in younger individuals, 14-3-3 has the same sensitivity compared to older individuals. 14-3-3 may be a useful adjunct when interpreting negative RT-QuIC results in younger individuals with suspected sCJD. RT-QuIC sensitivity in younger individuals may reflect an increased prevalence of rarer molecular subtypes associated with false negatives such as VV1 and MM2 [9].

Similar to findings in previous studies, our results suggest a low diagnostic sensitivity of EEG [10–12]. The presence of periodic triphasic complexes is not specific for sCJD and may be present in a wide array of neurological conditions [40]. Clinicians should be cautious when interpreting EEG findings in patients with suspected prion disease. A negative EEG should not falsely reassure against sCJD.

Boesenberg and colleagues previously suggested that the VV genotype may have a higher prevalence in the young [11]. We found that the distribution of codon 129 genotypes did not change with the age of onset, which is congruent with recent findings that indicate the mean age of onset is the same across codon 129 genotypes in a multi-national cohort [2].

In healthy populations, codon 129 polymorphism distribution is known to differ significantly between ethnic groups. Our study population is predominantly of white European descent. [41] Age-related differences in codon 129 polymorphism distribution in sCJD may be present in other ethnic groups.

### Neuropathological profiles

We did not identify significant neurodegenerative co-pathologies in patients that underwent post-mortem in our young age of onset group. In a literature-based case series of 20 sCJD individuals with an age of onset below 30, Corato and colleagues identified 3/17 individuals with amyloid plaque [10]. The size of our post-mortem group is small, but this finding is in keeping with the assumption that the brain of younger individuals would lack significant age-related neurodegenerative pathologies. sCJD was likely the sole neurodegenerative pathology contributing to their final illnesses. Boesenberg and colleagues quantified their sCJD lesion profiles, and suggested a greater degree of spongiform change, neuronal loss and gliosis in younger MM1 patients. The reverse was seen in VV2 patients. They did not report on the presence of any neurodegenerative co-pathologies.

The autopsy rate has declined in recent years in line with the increased sensitivity of current in-life diagnostic criteria [2, 25]. However, neuropathological examination remains important in individuals with atypical presentations and investigation results for surveillance of acquired and novel human prion diseases [7].

Age-related differences between molecular subtypes in sCJD have long been proposed [24, 42]. The presence of the rare VV1 subtype in younger age groups has been of particular interest [10, 11, 32]. Meissner and colleagues presented the largest case series of VV1 patients to date, presenting 9 individuals with an age of onset between 19 and 55 [32]. Parchi and colleagues identified 3 VV1 individuals in a cohort of 300 in their original characterisation of molecular classification in sCJD (age of onset 24–49) [24]. In Corato and colleagues’ literature-based case series, 6 VV1 individuals were present out of 8 with molecular subtype data (age of onset 17–29).

The VV1 molecular subtype remains rare in sCJD, but its hypothesised increased prevalence in younger individuals may underlie the different clinical and investigational characteristics we have outlined. They may be of clinical significance when assessing suspected sCJD in young individuals.

### Differential diagnosis

Our referral data showed 47% (41/87) of CJD cases referred to the NCJDRSU were eventually diagnosed with a prion disease other than sCJD in this age group. This highlights the importance of considering other subtypes of CJD in the assessment of younger individuals suspected of sCJD. In particular, vCJD is associated with younger age of onset in the third decade of life compared to sCJD [25]. Early psychiatric symptoms are common before the onset of cognitive and motor dysfunction, and disease duration is prolonged with median survival at 14 months [25, 43]. Our study, and findings from previous work, demonstrate similarities between the clinical features of vCJD and young-onset sCJD. [10, 11] In particular, Mok and colleagues described the most recent case of vCJD in the UK, where a pre-mortem diagnosis of probable sCJD was maintained until vCJD was neuropathologically confirmed on the post-mortem [29]. The individual did not meet the diagnostic criteria for vCJD in-life. The authors hypothesised whether this marked the start of a second wave of vCJD. This case was unique amongst vCJD in its heterozygosity at *PRNP* codon 129. There is potential for new atypical phenotypes of vCJD that more closely mimic the pre-mortem features of sCJD, although this has so far not materialised. Our data highlights the distinct diagnostic investigation profile of young-onset sCJD, and supports the use of MRI and CSF diagnostic tests to diagnose sCJD pre-mortem using current diagnostic criteria. However, the current situation also reinforce the importance of post-mortem examination in suspected prion disease, and additional vigilance for CJD surveillance programmes worldwide in the assessment of younger individuals.

Inherited prion disease is also an important consideration in younger age groups. There is a high degree of heterogeneity in the clinical features, age of onset and disease duration of inherited prion disease [44], which may mimic sCJD. *PRNP* sequencing should be routinely pursued to exclude inherited prion diseases in the diagnosis of sCJD.

Although very rare, the iatrogenic transmission of CJD remains a significant public health concern worldwide. Accurate diagnosis and comprehensive surveillance for all types of CJD is important in maintaining vigilance for acquired or novel forms of prion disease.

Several non-prion neurological conditions are known to mimic the presentation of sCJD [45], a number of which are important and commoner considerations in younger individuals. Amongst the seven individuals identified, in common were marked clinical and radiological improvement after referral, in contrast with the commonly rapid and progressive disease course seen in CJD. These individuals also had non-supportive neuroimaging and CSF biomarkers, highlighting the utility of these investigations and comprehensive assessment in young individuals presenting with rapidly progressive cognitive, psychiatric, and motor dysfunction.

### Care planning in young-onset sCJD

The UK NCJDRSU hosts the NHS England-funded National CJD Care Team, which provides post-diagnostic and longer-term support and guidance to people with sCJD and their families (M.L., T.H.). Our study findings are in keeping with our clinical experience of care coordination for younger individuals with sCJD. There are attendant complex and difficult care issues in this group; individuals are often of working age, with ongoing care responsibilities to family and dependents. The increased prevalence of psychiatric disturbance and personality changes often contributes to significant distress. The increased disease duration contributes to advanced care needs, with challenging gaps in services that are often bespoke to older people’s social care and end-of-life provisions.

### Experimental treatment

There is currently no disease-modifying treatment available for prion disease. For the majority of the study period there was no clinical trial registered in the UK for CJD. PRN100 was available in 2018 as a limited open-label series for 6 individuals under a Special License [46]. 5 of the individuals were diagnosed with sCJD, 3 of whom were included in our study. The focus of the study was to assess the safety and pharmacokinetics of the experimental treatment. Given the small sample size of the study, no significant overall survival or functional benefit (with MRC Prion Disease Rating Scale [47]) was identified to be statistically significant.

New experimental treatments are especially pertinent for young individuals with progressive and invariably fatal neurodegenerative conditions such as prion disease. Formal efficacy studies and clinical trials will further inform their potential use in clinical practice. The findings of our study may help improve diagnosis, which in turn may facilitate recruitment to future experimental treatment studies.

### Study strengths

Our study utilised a comprehensively annotated national surveillance cohort, with a high rate of case ascertainment and phenotyping. We identified a large group of 46 young individuals with sCJD, with well-documented clinical features and completed diagnostic investigations. This study provides a coherent narrative of the characteristics of sCJD in younger individuals, congruent with international findings from previous studies over the last 2 decades, and extends the current body of evidence by incorporating the impact of RT-QuIC analysis and diffusion-weighted MRI. Our study also provides a comparison to cases of other prion subtypes and non-prion diagnoses, further informing differential diagnosis in this group.

### Study limitations & future work

International CJD diagnostic criteria were updated in 2017 following the cooperation of CSF RT-QuIC and cortical ribboning on MRI brain. The authors acknowledge that there may have been a small degree of under-ascertainment in the earlier years of the study period prior to these developments. Within the young age of onset group, we identified only one case of possible sCJD between 31st August 2011 and 1st January 2017, which did not meet the criteria for probable sCJD based on the updated 2017 diagnostic criteria. The change in ascertainment level due to diagnostic criteria update is likely to be small.

Our data on symptoms is limited to two points in time: at illness onset and at the point of diagnosis. Longitudinal information regarding the onset of various symptoms may illustrate further age-related differences in sCJD symptomatology. Employment of validated symptom scoring systems in the longitudinal assessment of sCJD may help this by elucidating the evolution of symptoms in future studies of young-onset sCJD [47, 48].

Although age appears to be a major factor in disease duration in sCJD, other predictors such as codon 129 polymorphism also play a significant role. Prospective studies focused specifically on survival analysis, including confounders, would be valuable in providing more accurate prognostic information for young individuals with sCJD.

Due to the small autopsy sample size in the young age of onset group, we presented only limited data on the neuropathological profiles of younger individuals without statistical comparisons. Further autopsy case series with a larger number of individuals who undergo post-mortem may help to identify different neuropathological changes in young-onset sCJD.

## Conclusion

Our study complements the existing literature by providing a coherent narrative of the clinical, investigation and neuropathological characteristics of young-onset sCJD from a large national surveillance cohort. Our findings have important “real-world” clinical implications for early-onset cognitive services and prion disease surveillance programmes worldwide, informing early and accurate diagnosis of sCJD in young individuals. Considerations should be taken to exclude other prion disease subtypes, which continue to have significant public health implications worldwide. Improvements in the diagnosis of this group will aid effective care planning and support recruitment to future clinical trials in human prion disease.

## Supplementary Information

Below is the link to the electronic supplementary material.

Supplementary file1 (PDF 85 KB)

## Data Availability

The data that support the findings of this study are available on request from the corresponding author, S.P.

## References

[1] Uttley L, Carroll C, Wong R (2020). Creutzfeldt-Jakob disease: a systematic review of global incidence, prevalence, infectivity, and incubation. Lancet Infect Dis.

[2] Watson N, Hermann P, Ladogana A (2022). Validation of revised international Creutzfeldt-Jakob disease surveillance network diagnostic criteria for sporadic Creutzfeldt-Jakob disease. JAMA Netw Open.

[3] Ritchie D, Barria M (2021). Prion diseases: a unique transmissible agent or a model for neurodegenerative diseases?. Biomolecules.

[4] Rufai SB, Gupta A, Singh S (2019). Prion diseases: a concern for mankind. Pathogen Drug Resist Hum Pathogens Mech Novel Approach.

[5] Scott MR, Will R, Ironside J (1999). Compelling transgenetic evidence for transmission of bovine spongiform encephalopathy prions to humans. Proc Natl Acad Sci.

[6] Ward HJT, Everington D, Cousens SN (2006). Risk factors for variant Creutzfeldt-Jakob disease: a case–control study. Ann Neurol.

[7] Watson N, Brandel J-P, Green A (2021). The importance of ongoing international surveillance for Creutzfeldt-Jakob disease. Nat Rev Neurol.

[8] Vitali P, MacCagnano E, Caverzasi E (2011). Diffusion-weighted MRI hyperintensity patterns differentiate CJD from other rapid dementias. Neurology.

[9] Green AJE (2019). RT-QuIC: a new test for sporadic CJD. Pract Neurol.

[10] Corato M, Cereda C, Cova E (2006). Young-onset CJD: Age and disease phenotype in variant and sporadic forms. Funct Neurol.

[11] Boesenberg C, Schulz-Schaeffer WJ, Meissner B (2005). Clinical course in young patients with sporadic Creutzfeldt-Jakob disease. Ann Neurol.

[12] Shi Q, Xiao K, Chen C (2017). Clinical and laboratory features of 14 young Chinese probable sCJD patients. Prion.

[13] Maddox RA, Person MK, Schonberger LB (2016). Unusually young prion disease cases in the United States, 1979–2014. Prion.

[14] Lahiri D, Pattnaik S, Bhat A (2019). Young-onset sporadic Creutzfeldt-Jakob disease with atypical phenotypic features: a case report. J Med Case Rep.

[15] Appleby BS, Maddox R, Schonberger LB (2021). Sporadic Creutzfeldt-Jakob disease in a very young person. Neurology.

[16] Murray K, Ritchie DL, Bruce M (2008). Sporadic Creutzfeldt Jakob disease in two adolescents. J Neurol Neurosurg Psychiatry.

[17] Kulczycki J, Jedrzejowska H, Gajkowski K (1991). Creutzfeldt-Jakob disease in young people. Eur J Epidemiol.

[18] von Elm E, Altman DG, Egger M (2007). The Strengthening the Reporting of Observational Studies in Epidemiology (STROBE) statement: guidelines for reporting observational studies. The Lancet.

[19] National CJD Research & Surveillance Unit (2017) Protocol for Surveillance of CJD in the UK

[20] Creutzfeldt-Jakob Disease International Surveillance Network Diagnostic criteria for surveillance of CJD from 1 January 2017. In: 2017. https://www.eurocjd.ed.ac.uk/node/833. Accessed 8 Dec 2021

[21] Zerr I, Kallenberg K, Summers DM (2009). Updated clinical diagnostic criteria for sporadic Creutzfeldt-Jakob disease. Brain.

[22] Watson N, Kurudzhu H, Green A (2021). Application of telehealth for comprehensive Creutzfeldt-Jakob disease surveillance in the United Kingdom. J Neurol Sci.

[23] Steinhoff BJ, Zerr I, Glatting M (2004). Diagnostic value of periodic complexes in Creutzfeldt-Jakob disease. Ann Neurol.

[24] Parchi P, Giese A, Capellari S, et al (1999) Classification of sporadic Creutzfeldt-Jakob disease based on molecular and phenotypic analysis of 300 subjects. 10.1002/1531-8249(199908)46:2 10443888

[25] The National CJD Research & Surveillance Unit (2020) 29th Annual Report 2020—Creutzfeldt-Jakob Disease Surveillance in the UK

[26] R Core Team (2021) R: A language and environment for statistical computing

[27] Therneau T (2021) A Package for Survival Analysis in R

[28] Kassambara A, Kosinski M, Biecek P (2021) survminer: Drawing Survival Curves using “ggplot2”

[29] Mok T, Jaunmuktane Z, Joiner S (2017). Variant Creutzfeldt-Jakob disease in a patient with heterozygosity at PRNP CODON 129. N Engl J Med.

[30] Appleby BS, Appleby KK, Rabins Pv (2007). Does the presentation of Creutzfeldt-Jakob disease vary by age or presumed etiology? A meta-analysis of the past 10 years. J Neuropsychiatry Clin Neurosci.

[31] Stovner LJ, Hagen K, Linde M, Steiner TJ (2022). The global prevalence of headache: an update, with analysis of the influences of methodological factors on prevalence estimates. J Headache Pain.

[32] Meissner B, Westner IM, Kallenberg K (2005). Sporadic Creutzfeldt-Jakob disease: clinical and diagnostic characteristics of the rare VV1 type. Neurology.

[33] Pocchiari M (2004). Predictors of survival in sporadic Creutzfeldt-Jakob disease and other human transmissible spongiform encephalopathies. Brain.

[34] Chen C, Wang J-C, Shi Q (2013). Analyses of the survival time and the influencing factors of chinese patients with prion diseases based on the surveillance data from 2008–2011. PLoS ONE.

[35] Nagoshi K, Sadakane A, Nakamura Y (2011). Duration of prion disease is longer in Japan than in other countries. J Epidemiol.

[36] Sanchez-Juan P, Sánchez-Valle R, Green A (2007). Influence of timing on CSF tests value for Creutzfeldt-Jakob disease diagnosis. J Neurol.

[37] Sanchez-Juan P, Green A, Ladogana A (2006). CSF tests in the differential diagnosis of Creutzfeldt-Jakob disease. Neurology.

[38] Rhoads DD, Wrona A, Foutz A (2020). Diagnosis of prion diseases by RT-QuIC results in improved surveillance. Neurology.

[39] McGuire LI, Peden AH, Orrú CD (2012). Real time quaking-induced conversion analysis of cerebrospinal fluid in sporadic Creutzfeldt-Jakob disease. Ann Neurol.

[40] Hartshorn JA, Foreman B (2019). Generalized periodic discharges with triphasic morphology. J Neurocrit Care.

[41] Chen C, Dong XP (2016). Epidemiological characteristics of human prion diseases. Infect Dis Poverty.

[42] Hill AF, Joiner S, Wadsworth JDF (2003). Molecular classification of sporadic Creutzfeldt-Jakob disease. Brain.

[43] Heath CA, Cooper SA, Murray K (2010). Validation of diagnostic criteria for variant Creutzfeldt-Jakob disease. Ann Neurol.

[44] Mead S (2006). Prion disease genetics. Eur J Hum Genet.

[45] Mead S, Rudge P (2017). CJD mimics and chameleons. Pract Neurol.

[46] Mead S, Khalili-Shirazi A, Potter C (2022). Prion protein monoclonal antibody (PRN100) therapy for Creutzfeldt-Jakob disease: evaluation of a first-in-human treatment programme. Lancet Neurol.

[47] Thompson AGB, Lowe J, Fox Z (2013). The Medical Research Council Prion Disease Rating Scale: a new outcome measure for prion disease therapeutic trials developed and validated using systematic observational studies. Brain.

[48] Nihat A, Mok TH, Odd H (2022). Development of novel clinical examination scales for the measurement of disease severity in Creutzfeldt-Jakob disease. J Neurol Neurosurg Psychiatry.

