# Supplementary material for: Sporadic Creutzfeldt–Jakob Disease in the young (50 and below): 10-year review of United Kingdom surveillance
Source: J Neurol. 2022 Nov 5;270(2):1036–46. doi: 10.1007/s00415-022-11467-3 (PMC9886636; doi:10.1007/s00415-022-11467-3)
Supplement: Supplementary file 1 — Supplementary file1 (PDF 85 KB) [file 415_2022_11467_MOESM1_ESM.pdf]

Supplementary Materials

Presenting Symptom Category	Symptoms
Psychiatric & Behavioural Disturbance	depression, anxiety, paranoia, apathy, withdrawal, visual and auditory hallucinations, delusions, personality and behavioural changes
Cognitive Impairment	disorientation, attention deficit, memory impairment, navigational difficulties, dyscalculia
Motor & Gait Abnormalities	unsteadiness, clumsiness, incoordination, slowed movements, involuntary movements, tremors, myoclonus
Speech Disturbance	dysarthria, dysphonia, mutism
Language Disturbance	dysphasia, word finding difficulties, dyslexia
Visual Disturbances	diplopia, visual blurring, visual impairment, cortical blindness, visual illusions
Sensory Disturbance	numbness, tingling, pain (other than headache)
Auditory Disturbance	tinnitus, hearing difficulties
Headache	tension-type headache, migrainous headache
Sleep Disturbance	insomnia, parasomnia, REM sleep disorder
Dizziness & Vertigo	vertigo, light-headedness, dizziness
Fatigue & Malaise	-
Seizures	-
Other	sinus congestion, coughing

Supplement 1 –Symptom category definitions
